# Economic impacts of ambient ozone pollution on wood production in Italy

**DOI:** 10.1038/s41598-020-80516-6

**Published:** 2021-01-08

**Authors:** Sandro Sacchelli, Elisa Carrari, Elena Paoletti, Alessandro Anav, Yasutomo Hoshika, Pierre Sicard, Augusto Screpanti, Gherardo Chirici, Claudia Cocozza, Alessandra De Marco

**Affiliations:** 1 Department of Agriculture, Food, Environment and Forestry, P.le delle Cascine 18/via San Bonaventura 13, Florence, Italy; 2 IRET-CNR, Via Madonna del Piano 10, Sesto Fiorentino, Italy; 3 ENEA, Casaccia, Rome, Italy; 4 ARGANS, 260 route du Pin Montard, Sophia-Antipolis, France

**Keywords:** Environmental impact, Plant sciences

## Abstract

Worldwide, tropospheric ozone (O_3_) is a potential threat to wood production, but our understanding of O_3_ economic impacts on forests is still limited. To overcome this issue, we developed an approach for integrating O_3_ risk modelling and economic estimates, by using the Italian forests as a case study. Results suggested a significant impact of O_3_ expressed in terms of stomatal flux with an hourly threshold of uptake (Y = 1 nmol O_3_ m^−2^ leaf area s^−1^ to represent the detoxification capacity of trees), i.e. POD1. In 2005, the annual POD1 averaged over Italy was 20.4 mmol m^−2^ and the consequent potential damage ranged from 790.90 M€ to 2.85 B€ of capital value (i.e. 255–869 € ha^−1^, on average) depending on the interest rate. The annual damage ranged from 31.6 to 57.1 M€ (i.e. 10–17 € ha^−1^ per year, on average). There was also a 1.1% reduction in the profitable forest areas, i.e. with a positive Forest Expectation Value (FEV), with significant declines of the annual national wood production of firewood (− 7.5%), timber pole (− 7.4%), roundwood (− 5.0%) and paper mill (− 4.8%). Results were significantly different in the different Italian regions. We recommend our combined approach for further studies under different economic and phytoclimatic conditions.

## Introduction

Tropospheric ozone (O_3_) pollution affects large areas of the world^1,2^. Ozone is strongly phytotoxic and is considered as a serious issue for the health and productivity of forests^3^. Ozone risk assessment may use different O_3_ metrics^4^ or models^1^. The most common approach is the use of exposure-based metrics e.g. AOT40 i.e. the accumulation of hourly O_3_ concentrations above 40 ppb for daylight hours during the growing season^4^. However, exposure-based metrics do not incorporate the effects of stomata, that are the only way of O_3_ entry into the plant^5^. Therefore, a flux-based approach is recommended e.g. by the Convention on Long Range Transboundary Air Pollution of the United Nations^6^ and by the National Emission Ceilings Directive of the European Union^7^, where the stomatal O_3_ uptake is estimated through models integrating the effects of climatic factors and vegetation characteristics on stomata (e.g. the DO3SE model^8^). Such flux metric is called Phytotoxic Ozone Dose, defined as the amount of O_3_ absorbed into the leaves or needles through stomata over the growing season, and above a threshold Y of uptake (PODY).

Among the many ecosystem services provided by forests, only wood production losses have been estimated so far to assess the economic impact of O_3_ pollution on forests^9–11^, because experimental dose–response relationships are available for estimating biomass losses^12–14^. Feng et al.^11^ used the AOT40-response relationship and reported that current levels of O_3_ across China may cause economic losses of forest production equivalent to 52.2 billion US$ in 2015. Karlsson et al.^9^ similarly applied the AOT40-based approach to Swedish forests and simulated that the potential annual economic loss of forest production due to O_3_ was 56.0 million € over the time period 1993–2003. Felzer et al.^10^ predicted the O_3_ effects on carbon sequestration in crops, pastures and forests at global scale by using the empirical function of AOT40 for gross primary production (GPP) and found that reduced CO_2_ uptake due to O_3_ exposure would increase the macroeconomic consumption cost of the greenhouse gas policy by 4.5 trillion US$ in 2100. Wood is central in a sustainable bioeconomy, e.g. forestry and extended wood-based value chains employed 4.5 million people in the European Union in 2018^15^.

Given the limited knowledge on the economic value of O_3_-induced wood production losses, in particular according to a flux-based approach, the aim of this study was to develop a combined modelling approach for realistic estimates of the economic impacts of POD1-based wood losses, by using Italy as a case study. Italy is a well-known hot-spot of O_3_ pollution, given its central position in the Mediterranean basin in Southern Europe where climate and economic conditions promote O_3_ pollution^16^^.^^17^. In 2001, the Italian wood sectors employed 413,872 workers in 87,546 companies with a total turnover of about $35 billion, with the furniture sector accounting for $20.8 billion^18^. We expect that this approach and results stimulate further studies by using a harmonized methodology for a better economic understanding of the global impacts of O_3_ pollution on forests and the forestry sector.

## Results

**Figure 1:**
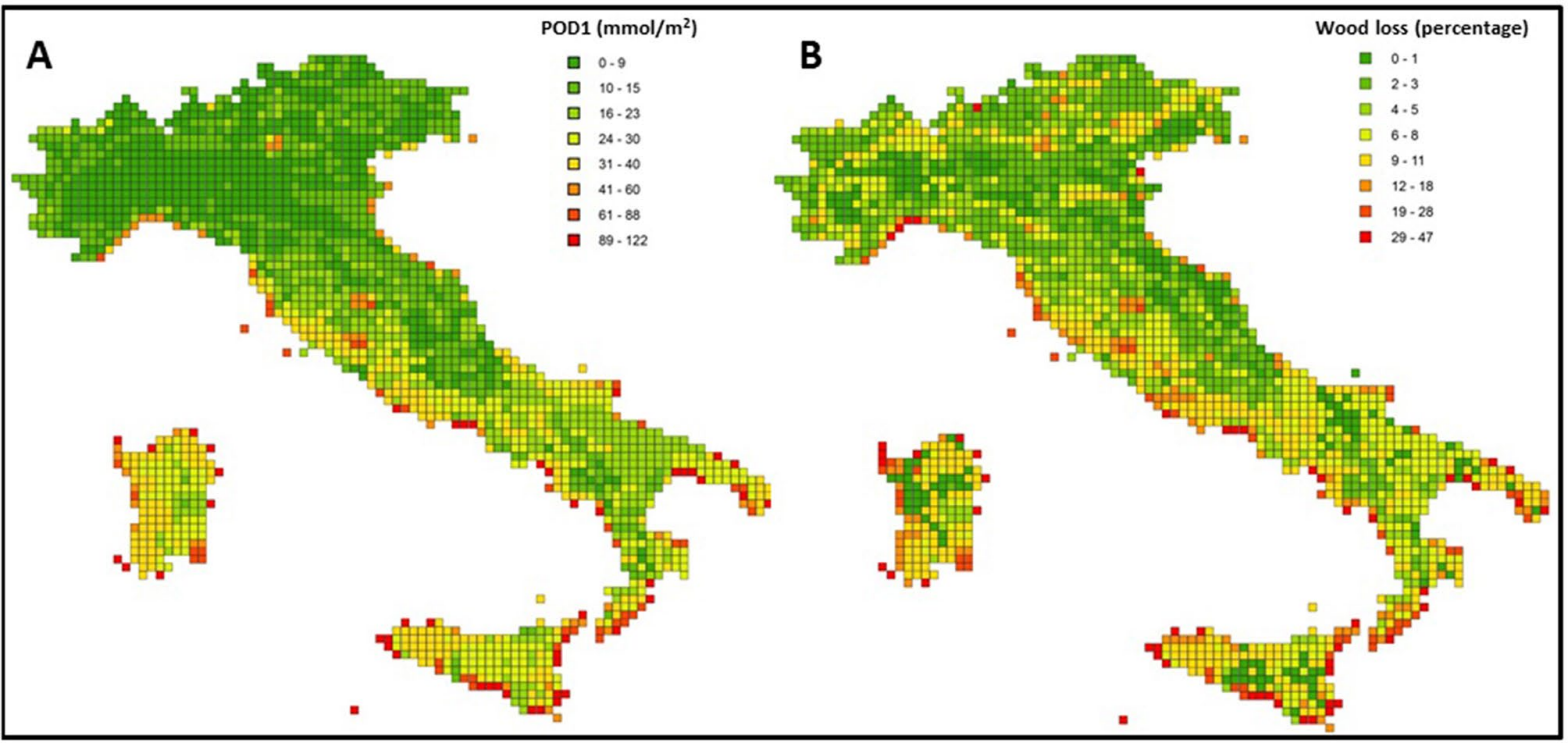
(**A**) Stomatal ozone uptake over a threshold of 1 nmol O_3_ m^−2^ s^−1^ (POD1) and (**B**) wood loss (%) in the POD1 scenario relative to the no ozone damage scenario (WO) estimated over a 12 × 12 km grid along Italy in the year 2005. Maps were created by QGIS (https://www.qgis.org/it/site/).

POD1 values across Italy ranged between 0.3 and 100 mmol m^−2^, with a spatial distribution showing lower values in the Alps mountains in the North and higher values over the peninsula and the islands where the climate is typically Mediterranean (Fig. 1A). The average value of POD1 was 20.4 mmol m^−2^ ranging from 8.0 mmol m^−2^ in Piedmont to 41.4 mmol m^−2^ in Sardinia (Fig. 1S).

Results suggested a significant impact of O_3_ (POD1) on Italian wood losses, that reached almost half of the expected wood production in the no ozone scenario (WO) at individual grid points (Fig. 1B) as well as a significant influence of the interest rate (r) on economic trend. Indeed, the total capital value of Italian forests in the WO scenario (no ozone) ranged from 8.0 to 29.5 B€ and from 2578 to 8987 € ha^−1^ with 2–4% of interest rate (Table 1). The POD1 scenario caused a total potential damage from 790.9 M€ to 2.85 B€ of capital value (255–869 € ha^−1^, on average). The annual damage ranged from 31.6 to 57.1 M€ (10–17 € ha^−1^ year^−1^, on average). The relative economic impact from WO to POD1 scenario was about 10% (total damage) and about 9% (average damage) for all interest rates. In addition, the total forest area with FEV > 0 decreased by around 1.1% from WO to POD1 (Table 2). The reduction ranged from 35,358 ha with r = 3% to 37,774 ha with r = 4% (1.1% and 1.2% of total forest area, respectively).

**Table 1:** Forest expectation value (FEV) under no ozone damage scenario (WO) and with a forest-type specific reduction in the annual forest increment as estimated on the basis of the phytotoxic ozone dose (POD1).

Scenario-FEV	Economic metrics	r = 2%	r = 3%	r = 4%
Scenario WO	Total (× 1000€)	29,515,620	13,698,530	7,988,405
	Average (€ ha^−1^)	8987	4293	2578
	Total annualized value (€ year^−1^)	590,312,400	410,955,900	319,536,200
	Average annualized value (€ ha^−1^ year^−1^)	180	129	103
Scenario POD1	Total (× 1000€)	26,662,800	12,356,890	7,197,459
	Average (€ ha^−1^)	8210	3916	2352
	Total annualized value (€ year^−1^)	533,256,000	370,706,700	287,898,360
	Average annualized value (€ ha^−1^ year^−1^)	164	117	94
FEV decrease from WO to POD1	Total (× 1000€)	2,852,820	1,341,640	790,946
	Average (€ ha^−1^)	869	420	255
	Total annualized value (€ year^−1^)	57,056,400	40,249,200	31,637,840
	Average annualized value (€ ha^−1^ year^−1^)	17	13	10
FEV decrease from WO to POD1	Total (%)	9.7%	9.8%	9.9%
	Average (%)	8.6%	8.8%	8.8%
	Total annualized value (%)	9.7%	9.8%	9.9%
	Average annualized value (%)	8.6%	8.8%	8.8%

Results are for the Italian forests in 2005, with different interest rates r.

**Table 2:** Total forest area (ha) with FEV > 0 and its decrease under no ozone damage scenario (WO) and phytotoxic ozone dose (POD1) scenario.

Total forest area with FEV > 0	r = 2%	r = 3%	r = 4%
Scenario WO (ha)	3,284,092	3,190,924	3,098,154
Scenario POD1 (ha)	3,247,741	3,155,566	3,060,380
FEV decrease from WO to POD1 (ha)	36,351	35,358	37,774
FEV decrease from WO to POD1 (%)	1.1%	1.1%	1.2%

Results are for the Italian forests in 2005, with different interest rates r.

The O_3_ impact on potential national wood production is reported in Table 3. According to the forest characteristics of Italy (widespread broadleaved stands, coppice management, private properties, etc.), the most represented wood assortment was firewood (from 5,449,258 to 5,603,228 m^3^ year^−1^ in WO scenario), followed by timber pole (from 818,390 to 824,274 m^3^ year^−1^), roundwood (from 587,011 to 672,446 m^3^ year^−1^) and paper mill (from 288,511 to 365,174 m^3^ year^−1^). Firewood and timber pole were strongly affected by moving from WO to POD1 scenario (on average the reduction was 7.5% and 7.4%, respectively). The decrease of roundwood and paper mill (on average 5.0% and 4.8%, respectively) was lower than that for timber pole and firewood.

**Table 3:** Timber production under no ozone damage scenario (WO) and phytotoxic ozone dose (POD1) scenario in Italy in 2005 and potential losses, with different interest rates r.

Scenario	Wood assortment	r = 2%	r = 3%	r = 4%
Scenario WO (m^3^ year^−1^)	Roundwood	672,446	633,599	587,011
	Timber pole	824,274	821,480	818,390
	Paper mill	365,174	328,267	288,511
	Firewood	5,603,228	5,525,739	5,449,258
Scenario POD1 (m^3^ year^−1^)	Roundwood	638,358	601,818	557,872
	Timber pole	762,918	760,319	757,445
	Paper mill	347,307	312,624	275,093
	Firewood	5,181,820	5,111,302	5,041,343
Loss from WO to POD1 (m^3^ year^−1^)	Roundwood	34,088	31,781	29,139
	Timber pole	61,356	61,161	60,945
	Paper mill	17,867	15,643	13,418
	Firewood	421,408	414,437	407,915
	Total	534,719	523,022	511,417
Loss from WO to POD1 (%)	Roundwood	5.1%	5.0%	5.0%
	Timber pole	7.4%	7.4%	7.4%
	Paper mill	4.9%	4.8%	4.7%
	Firewood	7.5%	7.5%	7.5%
	Average	6.2%	6.2%	6.1%

The Italian administrative regions were affected by O_3_ in different ways (Table 4). Total damage was affected more by the forest surface than by the average POD1 value of the region, i.e. was higher in regions with higher forest areas (from 1.03 M€ in the small Aosta Valley to 191.4 M€ in the big and highly forested Tuscany, equivalent to 0.03 and 5.70 M€ year^−1^, respectively). The average capital value showed higher impacts in Liguria (1229 € ha^−1^), Campania (628 € ha^−1^), Calabria (568 € ha^−1^) and Lazio (527 € ha^−1^) i.e. 37, 19, 17 and 16 € ha^−1^ year^−1^, respectively. The reduction of profitable forest area, i.e. with FEV > 0, was strongly affected by the POD1 level, and was higher in Sardinia (− 10,752 ha of forest surface area, 41.4 mmol m^−2^ POD1), followed by Calabria (− 5811 ha, 33.2 mmol m^−2^), Sicily (− 3362 ha, 40.9 mmol m^−2^), and then by highly forested regions with relatively lower POD1 levels such as Tuscany (− 2432 ha, 20.6 mmol m^−2^) and Trentino-South Tyrol (− 2319 ha, 9.2 mmol m^−2^). In relative terms, the decrease of profitable forests still occurred in Sardinia (6.2%), Sicily (3.1%) and Calabria (2.5%) but also in regions with a limited forest coverage but high POD1 level (e.g. 2.9% of reduction in Apulia, 32.2 mmol m^−2^).

**Table 4:** Reduction of forest expectation value (FEV) and forest area from the WO to the POD1 scenario.

Administrative region (from North to South)	Forest area (ha) and forest coverage (%)	FEV decrease from WO to POD1 (€)	FEV decrease from WO to POD1 (€ ha^−1^)	Decrease of forest surface with FEV > 0 from WO to POD1 (ha)	Decrease of forest surface with FEV > 0 from WO to POD1 (%)
Piedmont	940,116 (37%)	111,584,377	421	2122	0.8
Aosta Valley	105,928 (32%)	1,033,377	120	197	2.3
Lombardy	665,703 (28%)	62,116,668	438	954	0.7
Trentino-South Tyrol	779,705 (57%)	15,603,706	97	2319	1.4
Veneto	446,856 (24%)	50,099,297	415	565	0.5
Friuli-Venezia Giulia	357,224 (45%)	35,020,870	409	1049	1.2
Liguria	375,134 (69%)	163,607,975	1229	435	0.3
Emilia Romagna	608,818 (28%)	73,212,285	294	644	0.3
Tuscany	1,151,539 (50%)	191,470,424	338	2432	0.4
Umbria	390,255 (46%)	63,015,131	462	256	0.2
Marche	308,076 (32%)	24,256,891	368	376	0.6
Lazio	605,859 (35%)	84,261,787	527	422	0.3
Abruzzo	438,590 (41%)	26,627,345	234	325	0.3
Molise	148,641 (33%)	33,567,015	438	285	0.4
Campania	445,274 (33%)	121,124,452	628	831	0.4
Apulia	179,094 (9%)	22,399,791	456	1407	2.9
Basilicata	356,426 (36%)	48,243,555	311	814	0.5
Calabria	612,931 (41%)	132,897,988	568	5811	2.5
Sicily	338,171 (13%)	58,191,732	543	3362	3.1
Sardinia	1,213,250 (50%)	23,299,207	134	10,752	6.2

Results are for Italian regions in 2005, with interest rate r = 3%

## Discussion

We merged an open-source add-on GIS software tool for estimating the economic value of forests^19^ with a classic O_3_ risk assessment approach^6,20^, and used forest inventory and WRF-CHIMERE outputs to spatially estimate the O_3_-induced wood losses in the year 2005. We used the accumulated stomatal O_3_ uptake (POD1) as a metric of O_3_ damage because it is considered as a better index than only O_3_ concentrations in the air^21^. However, estimating POD1 at fine scale is challenging as it requires hourly inputs^22^. For the first time, an economic valuation of wood losses was based on POD1 at high spatial horizontal resolution (12 km^2^).

Italy is known to be subject to elevated O_3_ pollution^16^ and is thus an ideal case study for applying this combined approach. In fact, the average POD1 value of Italy was 20.4 mmol m^−2^ with spikes up to 100 mmol m^−2^. Such values are higher than values estimated at individual forest stands in central and Northern Europe (Tatra mountains^23^, 14–16 mmol m^−2^; Southern Sweden^24^, ~ 18 mmol m^−2^) and similar to values simulated for East-Asian forests (continental and (sub)tropical forests^20^, 20–105 mmol m^−2^). Also East Asia is known as a hot-spot of O_3_ pollution^25^. The spatial distribution of POD1 along the Italian peninsula showed lower values in the Alpine forests in the North (on average 12 mmol m^−2^) and higher values in the typical Mediterranean climate of the South (on average 23 mmol m^−2^) and the islands (~ 41 mmol m^−2^). These values and the North-to-South increase are similar to what observed for the entire Europe^26^, as the warmer climate in the South stimulates O_3_ formation^27^. These POD1 values are well above the critical levels of 4 and 8 mmol m^−2^ identified by^28^ for the protection of deciduous broadleaves (birch and beech) and conifers (Norway spruce), respectively, stressing that most of Italian forests are exposed to severe O_3_ risks.

Modelling O_3_ impact on Italian forests showed a marked decrease of their economic value. According to applied interest rate, annual damage ranged from 31.6 to 57.0 M€ per year with a loss of capital value of about 10%. Among the few previous papers on the economic impact of O_3_ on forests, none used a methodology comparable with the present research [9–11]. However, Karlsson et al.^9^ indicated similar results as the potential annual economic loss for Sweden due to negative impacts of O_3_ on forest production was of the order of 56 M€ that is about 2.4 € ha^−1^ year^−1^, while in Italy it ranged from 3.02 to 5.46 € ha^−1^ year^−1^ (r: 2–4%). The variability of potential economic impact among Italian administrative regions reflected the strong dissimilarity of geomorphological, logistic, vegetational as well as socio-economic conditions of Italian forests^29^. We innovatively defined the economic damage by O_3_ also in terms of reduction of forest area with positive FEV. This reduction in the POD1 scenario was about 1.1–1.2% of the total forest area. This loss of stands with economic profitability may make active forest management no longer meaningful, which would result in an indirect negative effect due to the worsening of other ecosystem services^30^. As an example, hydrogeological problems or fire risk—which are relevant issues for the Italian territory—can increase due to a decline of silvicultural practices^31^. In general, trade-offs among provisioning and other forest benefits (regulating, supporting and cultural services^32^) can happen and they may be measured not only in biophysical but also in economic terms^33^. A POD1-induced loss from about 535,000 to 511,000 m^3^ year^−1^ of wood assortments was also estimated. Firewood was the most impacted product (from 421,408 to 407,915 m^3^ year^−1^ of reduction) but the structure of Italian forest chain and the high added value for the other timber outputs (e.g., roundwood) suggested a potential negative cascade effect on the whole forest chain and on ancillary activities.

## Conclusions

This work is one of the first fine-scale combined models to quantify the economic impacts of O_3_ at national level. Results highlighted that the reduction of forest area with active management is limited to the most severely O_3_ polluted areas, even though significant negative effects on timber production occur all across Italy. Consequences on other forest ecosystem services and socio-economic deterioration of the forest chain, such as occupational consequences and cascade effect on satellite activities, should be evaluated. This aspect indicates that the elevated economic impact (loss of capital and annual values of forest) here presented, is still an underestimation of the total losses.

The open source software facilitates replicability as well as sensitivity analysis. Spatial analysis can take into account local peculiarities, thus helping to improve models and results, and finally resulting into guidelines to cope with the potential negative impacts of O_3_ pollution on forests and the forestry sector. This GIS-based application is thus a valuable tool to quantify and localize potential negative impacts. Ozone damages can interfere with the vitality of species of plant communities, as well as that of the animals, fungi, bacteria and insects that live in close association with plants or in nearby soils^34^. Changes induced by O_3_ impact on many ecological processes, affecting ecosystem services, flows, goods and values. Further activities in the definition of ecosystem-scale models suitable to extrapolate effects of O_3_ on productivity of trees and entire ecosystems might be addressed to economically quantify also the loss of other ecosystem services such as biodiversity, resource allocation and/or seed production.

## Methodology

### Study area and forest data

Data of biomass availability were obtained from the pan-European map of forest biomass increment^35^. The map was validated using information from the most recent and free georeferenced Italian National Forest Inventory, i.e. for the year 2005^36^. In Italy, the total forested area was 10,467,533 ha in 2005. There was a certain variability of forest cover (in percentage) as well as vegetation, geomorphologic, logistics and socio-economic characteristics among regions (NUTS-2 level). Private forests accounted for 63.5% of the total, and coppice was the prevalent forest management (54.0%). The most common forest typology was broadleaved species (83.6% of total area), mainly oaks (23.9% of broadleaved, mostly *Quercus robur* and *Q. cerris*), as well as *Fagus sylvatica* (11.8%) and *Castanea sativa* (9%). Among the conifers (16.4% of total area), *Picea abies* prevailed (34.2%), in particular in the Alpine forests^36^.

### Modelling ozone pollution

Hourly O_3_ concentrations and hourly meteorological data needed to calculate POD1 (i.e. solar radiation, air temperature, relative humidity, soil water content, wind speed) were simulated over the domain at 12 × 12 km of horizontal resolution by the WRF-CHIMERE modelling system, as described in^26^. The O_3_ concentrations at 20–25 m above ground level (top of the canopy) provided by CHIMERE were used to calculate PODY. For PODY, a threshold Y of 1 nmol m^−2^ s^−1^ per leaf area as recommended by^6^ for forest protection was applied, and computed as in^6,37^:

1 $${\text{POD1}}\left( t \right) = \mathop \smallint \limits_{SGS}^{EGS} \max \left( {\frac{Rc}{{Rb + Rc}} \times g_{sto} \times \left[ {{\text{O}}_{3} } \right] - {1, 0}} \right)dt$$

where [O_3_] is hourly O_3_ concentrations (ppb), *dt* is time step (1 h), SGS and EGS are the start and end date of the growing season computed as described in^38^, R_b_ is the quasi-laminar resistance (s m^−1^), R_c_ is the leaf surface resistance (s m^−1^), and g_sto_ is the hourly value of stomatal conductance to O_3_ (mmol O_3_ m^−2^ PLA s^−1^, where PLA is the Projected Leaf Area) computed as following:

2 $$g_{sto} = g_{max} \times f_{phen} \times f_{light} \times max \left\{ {\left\{ {f_{min} } \right.,\left( {f_{temp} \times f_{VPD} \times f_{SWC} } \right.)} \right\}$$

where g_max_ is the maximum stomatal conductance to O_3_ of a plant species expressed on a total leaf surface area (mmol O_3_ m^−2^ PLA s^−1^). The maximum stomatal conductance (g_max_) is experimentally obtained as average above the 90^th^ or 98^th^ percentile of g_sto_ measurements under optimum environmental conditions for stomatal opening^6,14^. The functions *f*_phen_, *f*_light_, *f*_temp_, *f*_VPD_, and *f*_SWC_ are the variation in g_max_ with leaf age, photosynthetically flux density at the leaf surface (PPFD, μmol photons m^−2^ s^−1^), surface air temperature (T, °C), vapour pressure deficit (VPD, kPa), and volumetric soil water content (SWC, m^3^ m^−3^), respectively. The function *f*_min_ is the minimum stomatal conductance. These species-specific functions vary between 0 and 1, and are expressed as:

3 $$f_{light} = 1 - e^{{\left( { - light_{a} \times PPFD} \right)}}$$

4 $$f_{temp} = \left( {\frac{{T - T_{\min } }}{{T_{opt} - T_{\min } }}} \right)*\left[ {\left( {\frac{{T_{\max } - T}}{{T_{\max } - T_{opt} }}} \right)^{{\left( {\frac{{T_{\max } - T_{opt} }}{{T_{opt} - T_{\min } }}} \right)}} } \right]$$

5 $$f_{VPD} = \min \left\{ {1,\max \left[ {f_{\min } ,\left( {\frac{{(1 - f_{\min } )*(VPD_{\min } - VPD)}}{{VPD_{\min } - VPD_{\max } }}} \right) + f_{\min } } \right]} \right\}$$

6 $$f_{SWC} = \min \left[ {1,\max \left( {f_{\min } ,\frac{SWC - WP}{{FC - WP}}} \right)} \right]$$

where light_a_ is an adimensional constant; PPFD is hourly photosynthetic photon flux density estimated through the solar radiation; T_opt_, T_min_, and T_max_ represent the optimum, minimum, and maximum temperature for g_sto_, respectively; VPD_min_ and VPD_max_ are minimum and maximum VPD for g_sto_; and WP and FC are the soil water content (SWC) at wilting point and field capacity, respectively^6^. WP and FC are constant and depend on the soil type obtained from a module included into the WRF-CHIMERE model. We assumed that *f*_phen_ was 1 throughout the growing season (0 otherwise).

Six types of parameterization were used based on the six dominant forest types identified by land cover and climate over Italy, i.e. Alpine (for which the Boreal parameterizations in^6^ was used), Continental and Mediterranean with either Deciduous or Evergreen species (Table 5). After calculation of POD1, the dose–response functions specific per each forest type were applied in each 12 × 12 km grid point according to Table 6.

**Table 5:** Forest-type parameterization of DO3SE model according to^6^.

Biogeographic region	Alpine		Continental		Mediterranean	
Forest type	Evergreen	Deciduous	Evergreen	Deciduous	Evergreen	Deciduous
g_max_ (mmol O_3_ m^−2^ PLA s^−1^)	125	240	130	155	195	265
fmin (fraction of g_max_)	0.10	0.10	0.16	0.13	0.02	0.13
light_a (dl)	0.0060	0.0042	0.0100	0.0060	0.0120	0.0060
T_min_ (°C)	0	5	0	5	1	0
T_opt_ (°C)	20	20	14	16	23	22
T_max_ (°C)	200	200	35	33	39	35
VPD_max_ (kPa)	0.8	0.5	0.5	1.0	2.2	1.1
VPD_min_ (kPa)	2.8	2.7	3.0	3.1	4.0	3.1

g_max_, maximum stomatal conductance; *f*_min_ minimum stomatal conductance; *f*_light_a_ parameter determining the shape of the hyperbolic relationship of stomatal response to light (dimensionless); T_max_, T_opt_ and T_min_ are maximum, optimal and minimum temperature for calculating the function *f*_temp_ that expresses the variation of g_max_ with temperature; VPD_min_ and VPD_max_ are the vapor pressure deficit for attaining minimum and full stomatal aperture calculating the function *f*_VPD_ that expresses the variation of g_max_ with vapor pressure deficit. The parameters for the soil water content (*f*_SWC_) and phenological functions (*f*_phen_) are obtained by the WRF model and vary with the latitude.

**Table 6:** Forest-type dose–response functions to estimate total biomass losses (L) based on the cumulated stomatal ozone flux above a threshold of 1 mmol m^−2^ (POD1) according to^6^.

Forest type	Dose–response function
Alpine deciduous	L = 100.2 − (0.93*POD1)
Alpine evergreen	L = 99.8 − (0.22*POD1)
Continental deciduous	L = 100.2 − (0.93*POD1)
Continental evergreen	L = 99.8 − (0.22*POD1)
Mediterranean deciduous	L = 100.3 − (0.32*POD1)
Mediterranean evergreen	L = 99.8 − (0.09*POD1)

### Modelling ozone-induced wood losses

The economic value of Italian forests was quantified through a spatial-based analysis centered on the r.green.biomassfor model^39^. The tool is available as open-source add-on in GRASS Geographic Information System (GIS) software (https://grass.osgeo.org/grass78/manuals/addons/r.green.biomassfor.html). The model allows a quantification of wood assortments as well as their economic value by a multistep procedure. First, the so-called “technical availability” of material is quantified considering logistic (distance from forest/main roads or landing site) and geomorphological conditions (slope and terrain roughness). The combination of the above variables allows defining forest production process in terms of organization of cutting, processing and extraction. The second step introduces the economic parameters to calculate revenues from sell of assortments and full costs. Potentially available material is finally quantified on forest surfaces with economic profitability of production process. In the original version of the model, profitability was expressed as positive stumpage value (difference between revenues and costs of final harvesting / thinning). Here—in order to evaluate a long-term impact—the positive forest expectation value (FEV) (capital value of bare land plus timber at year *y*) was considered as index of economic profitability. FEV corresponds to the present value of cashflows arising from both the land and the tree, in perpetuity^40^. Input data to run r.green.biomassfor in Italy were derived from^19^ and are provided in Tables S1 and S2. The complete procedure was developed on raster basis with a resolution of 1 ha (squared pixel of 100 × 100 m). Wood losses due to O_3_ were computed taking into account the reduction of forest increment. In the present work, two scenarios were developed to quantify FEV and—as a consequence—forest surface with positive economic value and wood production: (i) a hypothetical scenario without O_3_ impact (WO) and (ii) a scenario based on POD1 limiting biomass production. The software r.green.biomassfor was then ran for both scenarios and results were reported at national and administrative (regions) level. FEV for each scenario *s* (FEVs) was calculated as in^41^ based on the “future revenues” approach:

7 $$FEV_{s} = \frac{{SV_{s} + \mathop \sum \nolimits_{m} T_{s} \cdot q^{{t_{s} - m_{s} }} + \left( {v_{s} - e_{s} } \right) \cdot \left( {\frac{{q^{{t_{s} - n_{s} }} - 1}}{r}} \right) + LEV_{s} }}{{q^{{t_{s} - n_{s} }} }}$$

where *SV* is stumpage value of final harvesting, *T* is stumpage value of intermediate thinning, *t* is rotation period, *m* is year of thinning, *n* is age of the forest, *v* and *e* are yearly income and cost, respectively, *q* = *1* + *r* with *r* interest rate, *LEV* is the land expectation value (bare soil) calculated as in^42^:

8 $$LEV_{s} = \frac{{SV_{s} + \mathop \sum \nolimits_{m} T_{s} \cdot q^{{t_{s} - m_{s} }} }}{{q^{{t_{s} }} - 1}} + \frac{{v_{s} - e_{s} }}{r}$$

FEVs were calculated at national and region level for both total (€) (Eq. 9) and average (€/ha) (Eq. 10) values. As POD1-based damage is accumulated over the growing season, total and average FEVs were annualized (aFEV) as expressed in Eq. (11) and Eq. (12), respectively.

9 $$FEV_{\omega } = \mathop \sum \limits_{i = 1}^{x} FEV_{i} \forall i \in \omega$$

10 $$averageFEV_{\omega } = \frac{{\mathop \sum \nolimits_{i = 1}^{x} FEV_{i} }}{{\mathop \sum \nolimits_{i = 1}^{x} i}} \,where\, i \in FEV > 0 \wedge \forall i \in \omega$$

11 $$aFEV_{\omega } = FEV_{\omega } \cdot r$$

12 $$average\_aFEV_{\omega } = averageFEV_{\omega } \times r$$

where *ω* is ω-th administrative level, *i* is i-th pixel in the map, the expression $i \in FEV > 0 \wedge \forall i \in \omega$ represents forest surfaces with positive FEV for ω-th administrative level, and r is interest rate.

In each scenario *s*, the amount of timber potentially obtained was calculated on the basis of forest surface with positive FEV, forest type and partitioning (percentage) of biomass increment in each wood assortment. The analysis of economic losses was focused on forest surfaces where FEV > 0 in WO scenario because damage evaluation must compare ex-ante (WO scenario) and ex-post (POD1) situations. Therefore, in the hypothesis of timber products investigation, an economic damage exists if and only if forest area can be economically processed also in WO scenario. The main assortments considered as typical for Italy are roundwood, timber pole, paper mill and firewood^19^.

Long-term economic valuation (that is typical in forest sector) is very variable relative to the interest rate applied for capitalization. Therefore, a sensitivity analysis based on variation of the interest rate *r* was performed at national level. In other terms, economic metrics (outputs, see Table 1) were computed for three different interest rates (2%, 3% and 4%) that are appropriate in Italy on the basis of forest and forest owners’ characteristics^41^. Sensitivity analysis facilitates depiction of potential range of results when input variables can cause high level of uncertainty in the outputs. Regional evaluation considered—for brevity—only r = 3%^43^ for quantification of FEV as well as forest surface with positive FEV.

To link biomass from dose response functions to economic losses, the O_3_-induced biomass reductions (POD1 scenario) were attributed to the forest map by overlaying spatial locations. The forest map was created based on Corine Land Cover (CLC) polygons (Table S1). Each CLC forest polygon reports one forest category and was linked to one biogeographical region of Italy (i.e. alpine, continental or Mediterranean^44^). The CLC polygons were associated, through specific alphanumeric rules, to the forest types applied to compute dose–response functions (alpine deciduous, alpine evergreen, continental deciduous, continental evergreen, Mediterranean deciduous, Mediterranean evergreen). In case of lack of spatial matching among CLC polygons and POD1 forest types, a geographical extension of POD1 information was performed by means of proximity analysis (i.e. Voronoi tassellation of POD1 centroids^45^). The results were finally mapped by QGIS (https://www.qgis.org/it/site/).

## Supplementary information

Supplementary Information 1.
