# Supplementary material for: Economic impacts of ambient ozone pollution on wood production in Italy
Source: Sci Rep. 2021 Jan 8;11:154. doi: 10.1038/s41598-020-80516-6 (PMC7794517; doi:10.1038/s41598-020-80516-6)
Supplement: Supplementary file 1 — Supplementary Information 1. [file 41598_2020_80516_MOESM1_ESM.docx]

**Supplementary material**

**Table S1.** Land Information System for FEV quantification in the r.green.biomassfor model^19^ modified and updated

| **Input variable** | **Description** | **Source** |
| --- | --- | --- |
| DTM | Digital Terrain Model | Italian National Geoportal, http://www.pcn.minambiente.it/mattm/servizi-di-scaricamento/ |
| Forest roads | Forest road features | Openstreetmap Project, (http://download.geofabrik.de/europe/italy.html |
| Forest typology | Corine Land Cover (CLC) map | Italian Institute for Environmental Research and Protection (ISPRA), http://www.sinanet.isprambiente.it/it/sia-ispra/download-mais/corine-land-cover |
| Forest increment | Annual increment (m^3^/ha) | Pan-European Map of Forest Biomass Increment (http://ies-ows.jrc.ec.europa.eu/efdac/download/ForestBiomassIncrement.zip). |
| Percentage of assortments | Partitioning of increment in different wood assortments | Based on CLC categories, analysis of local market and national norm tables from ISAFA, http://mpf.entecra.it/sites/default/files/pub_interne/ |
| Price of assortments | Selling price of assortments at landing site (€/m^3^) | Analysis of local Chambers of Commerce, Industry, Crafts and Agriculture; Wood Market analysis [Archivio Borsa Legno] of the Sherwood journal, http://www.rivistasherwood.it/extra/archivio-borsa-legno.html) |
| Forest management | 1: high forest, 2: coppice | Sacchelli et al., 2018 |
| Forest treatment | 1: final harvesting, 2: thinning | Sacchelli et al., 2018 |
| Lakes | Lake polygons | Italian National Geoportal, http://www.pcn.minambiente.it/mattm/servizi-di-scaricamento/ |
| Rivers | River lines | Italian National Geoportal, http://www.pcn.minambiente.it/mattm/servizi-di-scaricamento/ |
| Roughness | Roughness classification: 0: no rugged, 1: locally rugged, 2: partially rugged, 3: prevalently rugged | From r.green.biomassfor model |
| Mean tree diameter | Average diameter (cm) | From r.green.biomassfor model |
| Mean tree volume | Average single tree volume (m^3^) | From r.green.biomassfor model |
| Soil productivity | Soil fertility category: 1: very low, 2: low, 3: medium, 4; high, 5: very high | From r.green.biomassfor model |
| Protected areas | Boundary of national, regional and  provincial parks, national and provincial nature reserves, protected natural areas of local interest, as well as Natura 2000 network sites | Italian National Geoportal, http://www.pcn.minambiente.it/mattm/servizi-di-scaricamento/ |
| Boundary | Used to calculate output (national and regional boundaries) | Italian National Institute for Statistics (ISTAT), http://www.istat.it/it/archivio/124086 |

**Table S2.** Coefficients applied in the r.green.biomassfor model^19^

| **Variable** | **Value** |
| --- | --- |
| Interest rate (%) | Range 2% - 4% |
| Rotation period (years) | Varies according to forest typology, forest management and region |
| Age of thinning (years) | Applied rule:  if *t*<40 then *m*=10; if 40≤*t*<70 then *m*=15; if *t*≥70 then *m*=25 |
| Age of forest (years) | t/2 |
| Yearly income (€ ha^-1^ year^-1^) | 0 |
| Yearly costs (€ ha^-1^ year^-1^) | 20 |
| Regeneration cost (€ ha^-1^) | Costs of 1000 € ha^-1^ and 500 € ha^-1^ were considered respectively in coniferous and mixed/broadleaved forests, to cope with failed plantation/natural regeneration |

**
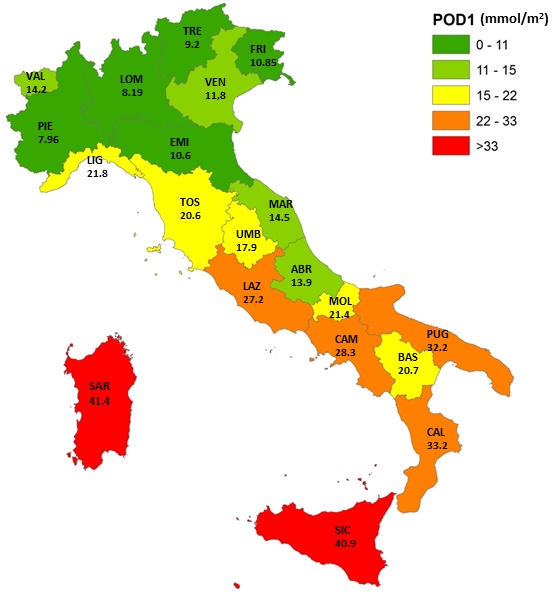
**

**Figure 1S.** Average POD1 values (mmol m^-2^) of the Italian administrative regions in 2005. The map was created by QGIS (https://www.qgis.org/it/site/).
